# Targeting the duality of cancer

**DOI:** 10.1038/s41698-017-0026-x

**Published:** 2017-06-22

**Authors:** Jack L. Arbiser, Michael Y. Bonner, Linda C. Gilbert

**Affiliations:** Department of Dermatology, Emory University School of Medicine, Atlanta Veterans Administration Medical Center, Winship Cancer Institute, Atlanta, GA USA

## Abstract

Cancer is the second leading cause of death in the United States, and is an increasing cause of death in the developing world. While there is great heterogeneity in the anatomic site and mutations involved in human cancer, there are common features, including immortal growth, angiogenesis, apoptosis evasion, and other features, that are common to most if not all cancers. However, new features of human cancers have been found as a result of clinical use of novel “targeted therapies,” angiogenesis inhibitors, and immunotherapies, including checkpoint inhibitors. These findings indicate that cancer is a moving target, which can change signaling and metabolic features based upon the therapies offered. It is well-known that there is significant heterogeneity within a tumor and it is possible that treatment might reduce the heterogeneity as a tumor adapts to therapy and, thus, a tumor might be synchronized, even if there is no major clinical response. Understanding this concept is important, as concurrent and sequential therapies might lead to improved tumor responses and cures. We posit that the repertoire of tumor responses is both predictable and limited, thus giving hope that eventually we can be more effective against solid tumors. Currently, among solid tumors, we observe a response of 1/3 of tumors to immunotherapy, perhaps less to angiogenesis inhibition, a varied response to targeted therapies, with relapse and resistance being the rule, and a large fraction being insensitive to all of these therapies, thus requiring the older therapies of chemotherapy, surgery, and radiation. Tumor phenotypes can be seen as a continuum between binary extremes, which will be discussed further. The biology of cancer is undoubtedly more complex than duality, but thinking of cancer as a duality may help scientists and oncologists discover optimal treatments that can be given either simultaneously or sequentially.

## Mutant p53 vs. loss of p16ink4a

P53^1^ and p16ink4a are the most common tumor suppressors lost in human cancer.^2–6^ We and others have shown that tumors with these mutations signal differently and, while this is well established in the world of basic research, it is not well appreciated by clinicians.^7–9^ It is well-known that certain cancer types have a large predominance of one type of tumor suppressor loss over another. For example, melanoma ranks among the top tumors for mutational burden and commonly does not express p16ink4a, but mutation of p53 is very uncommon in melanoma.^10–12^ On the other hand, cutaneous squamous cell carcinoma, one of the most common cancers in humans, more commonly has p53 mutation than loss of p16ink4a.^13–15^ Another observation is that while many tumors completely lack p16 expression, complete loss of p53 function is uncommon. This might be that having a functional p53 allele allows tumors to halt replication in response to DNA damage.^16, 17^

We first observed a signaling dichotomy between p53 and p16ink4a during our studies of angiosarcoma, a malignancy of endothelial cells. In the model we generated by sequential introduction of SV40 large T antigen and oncogenic H-ras, we noted that angiogenesis and in vivo growth was regulated by phosphoinositol-3 kinase (PI3K) signaling.^18^ This was the first observation that PI3K regulated in vivo growth and angiogenesis of a solid tumor.^18^ Since that time, activating mutations in PI3K signaling have been observed in a wide variety of human tumors. When we inhibited p42/44 MAP kinase signaling in these angiosarcoma cells, we noted slowed *in vitro* growth, but more aggressive in vivo growth and production of matrix metalloproteinases.^7^ Thus, in this system, MAP kinase was acting as a tumor suppressor, in contradiction to its well-known oncogenic activity in NIH3T3 fibroblasts. In order to understand the context difference in signaling, we noted that NIH3T3 fibroblasts have lost p16ink4a expression,^19^ while our angiosarcoma cells have defective p53 signaling.^18^ We thus hypothesized that p42/44 MAP kinase signaling is oncogenic in the context of loss of p16ink4a, but may be tumor suppressive in the mutant p53 context. In fact, this has been elegantly demonstrated accidentally in patients receiving the Braf inhibitor vemurafenib, which inhibits p42/44 MAPK signaling in melanoma, but can provoke squamous cell carcinoma, which has p53 mutations.^14^ The major discovered differences between these tumors have increased and are summarized in Table 1.

**Table 1:** Signaling differences and mutations in tumors that have either mutant p53 or loss of p16ink4a

Mutant p53	Loss of pl6
p42/44 MAPK possible tumor suppressor	p42/44 MAPK pro-tumorigenic
Negative for Wilms′ Tumor 1	Positive for Wilms′ Tumor 1
Ameboid morphology	Mesenchymal morphology
Lymph nodes	Hematogeneus
Radiation sensitive	Radiation resistant
Notch inactivate	Notch activation
Survivin	Superoxide
Stat 3	Stat 3/5
Hif1α	Hif2α
	Extracellular membrane deposition
	Increased telomerase

These represent polar opposites, but in some very advanced tumors, there may be both mutant p53 and loss of p16ink4a, leading to potential signaling plasticity

## Patterns of carcinogenesis

It is well-known that certain cancers are associated with high rates of p16ink4a loss, while others are associated with high rates of mutant p53. Among the tumors associated with loss of p16in4a and wild-type p53 are melanoma, primary glioblastoma, mesothelioma, ER/PR^20^ and her2/neu-positive breast carcinoma,^21^ bladder cancer associated with Schistosomiasis, some alcohol, and tobacco-associated head and neck cancers, some lung cancers, and inflammation-induced colon cancer,^22^ and virtually all cancers induced by Epstein–Barr virus (EBV) (Burkitt’s lymphoma, Hodgkins disease, gastric carcinoma, etc.) and hepatitis C virus (hepatocellular carcinoma), among others.^8, 23, 24^

Cancers associated with mutations in p53 include a majority of non-melanoma skin cancers, secondary glioblastoma associated with prior chemotherapy, other chemotherapy-induced malignancies (secondary leukemias, etc.), triple-negative breast carcinoma,^25, 26^ subsets of gastrointestinal malignancies, and subtypes of lung cancers, among others.^27^ Our research and others’ posit that different mutational stimuli are associated with differing mutational profiles. One early experiment showed that nickel sulfide, an oxidative carcinogen, caused sarcomas in mice with both 1 and 2 copies of functional p53. In all of these mice, hypermethylation of p16ink4a was a common feature.^8^ We postulated that hypermethylation of tumor suppressor genes, especially p16ink4a, is a canonical response to chronic oxidative stress. Later, it was discovered that reactive oxygen induces the enzyme responsible for hypermethylation of tumor suppressor genes, DNA methyltransferase 1.^28, 29^

Another example of oxidative carcinogenesis is that of the EBV, one of the most common viral carcinogens. EBV was initially discovered in African Burkitt’s lymphoma, a highly aggressive malignancy.^4^ Burkitt’s lymphoma also occurs in a sporadic form and, unlike EBV-associated Burkitt’s lymphoma, sporadic Burkitt’s lymphoma commonly exhibits mutant p53.^4^ We demonstrated that EBV-positive Burkitt’s lymphoma cells have elevated levels of reactive oxygen compared to EBV-negative cell lines.^9, 30^ The increased levels of reactive oxygen are mediated by EBV-specific genes, including EBNA2 and LMP1, and can be antagonized by the reactive oxygen inhibitor ebselen.^9^ Levels of vascular endothelial growth factor can be antagonized in EBV-positive cells by a p42/44 MAP kinase inhibitor, but not in EBV-negative cells with mutant p53. This finding clearly shows that context is important, and that p16ink4a null cells signal very differently than p53 mutant cells of the same tumor type. This knowledge has been used clinically, as gentian violet, a small molecular weight NADPH oxidase inhibitor,^31, 32^ has been found to be efficacious in treating oral hairy leukoplakia, an EBV infection of oral epithelium, in a HIV-positive patient.

Mutant p53 has been found in non-melanoma skin cancer, where it has been shown to occur as UV signature mutations, in which ultraviolet light b directly damages DNA.^33^ Mutations in p53 occur in non-random locations that affect some, but not all, functions of p53, therefore acting as dominant-negative mutations. It is not unreasonable to postulate that all pathogenic p53 mutations result from direct DNA damage from a mutagen, while loss of p16ink4a results from chronic oxidative stress, which may be due to ultraviolet A (melanoma), chronic inflammation (colon cancer, mesothelioma due to asbestos), viral oncogenesis (EBV), and other insults.^12, 34, 35^

As mentioned before, loss of p16ink4a and mutant p53 are associated with differing signaling pathways. In our previous studies with nickel sulfide-induced oxidative carcinogenesis, we found p42/44 MAP kinase activation in all tumors induced by nickel sulfide.^8^ Similarly, a majority of melanomas express mutations in Braf, which is upstream of MAP kinase, and we have shown that a majority of human melanomas express activated MAP kinase, regardless of mutational status.^36–38^ Finally, reactive oxygen can inactivate several tumor suppressor genes by oxidizing sulfhydryl groups, leading to coordinate inactivation of the tumor suppressor genes p53, PTEN and IκB.^39^ This leads to activation of tumor-promoting signaling of Akt and NFκB. Suppression of reactive oxygen can lead to activation of wild-type p53, inactivation of Akt, and NFκB.^40^ We have observed this in human melanoma in which application of the NADPH oxidase inhibitor gentian violet to an advanced melanoma led to a durable remission in an elderly patient.^41^ This inhibitor also downregulates Sox2, indicating that Sox2 downregulation might be a target of NADPH oxidases.^42^ Of interest, the solid tumors that are most responsive to chemotherapy, choriocarcinoma, and seminoma have very low levels of Sox2 expression, thus indicating that reduction of Sox2 might sensitize tumors to conventional chemotherapy.^43, 44^

## Lymph node vs. hematogenous metastasis

Tumors have a well-known propensity to metastasize and this property is the leading cause of death due to cancer, with local invasion being a less common cause. Distant metastasis of tumors to vital organs such as brain, lung, and liver cause death due to organ compromise. Lymph node metastasis occurs as well, and even occurs with some benign lesions such as Spitz nevi, melanocytic lesions with oncogenic mutations.^45, 46^ Clinical observations play a crucial role in elucidating the biology of metastasis. For example, in cutaneous melanoma, both lymph node metastasis and distant metastasis are common. In ocular melanoma, metastasis is invariably to the liver, due to the presence of trophic hepatocyte growth factor. In Merkel cell carcinoma, another aggressive carcinoma of the skin, metastases are mostly lymphatic.^47, 48^ Breast carcinoma metastasizes both to lymph nodes and distantly.^49^ The propensity to metastasize to different sites has given rise to the concept of lymphatic vs. hematogenous metastasis, and it is now well-known that tumor cells may condition their niche prior to metastasis to lymphatics, or distantly. Finally, tumor cells have been shown to migrate as either mesenchymal or ameboid.^50–52^ While all of this is known, it has not been tied together. We propose that different signaling pathways underlie distant metastasis vs. lymphatic metastasis. Moreover, the preponderance of published data suggests that distant hematogenous metastases are linked to mesenchymal migration, while lymphatic metastases are linked to ameboid migration.^53^ Finally, we believe that hematogenous metastasis is linked to reactive oxygen-rac signaling, while lymphatic metastasis is linked to alternative pathways, i.e., rhoA and rhoC.^54–60^ The clinical predominance of lymphatic vs. distant metastasis may be a marker of the plasticity of the tumor, with tumors that cause both lymphatic and distant metastasis demonstrating a high degree of plasticity, while tumors where there is a strong predominance of either lymphatic or distant disease have less signaling plasticity.

**Fig. 1:**
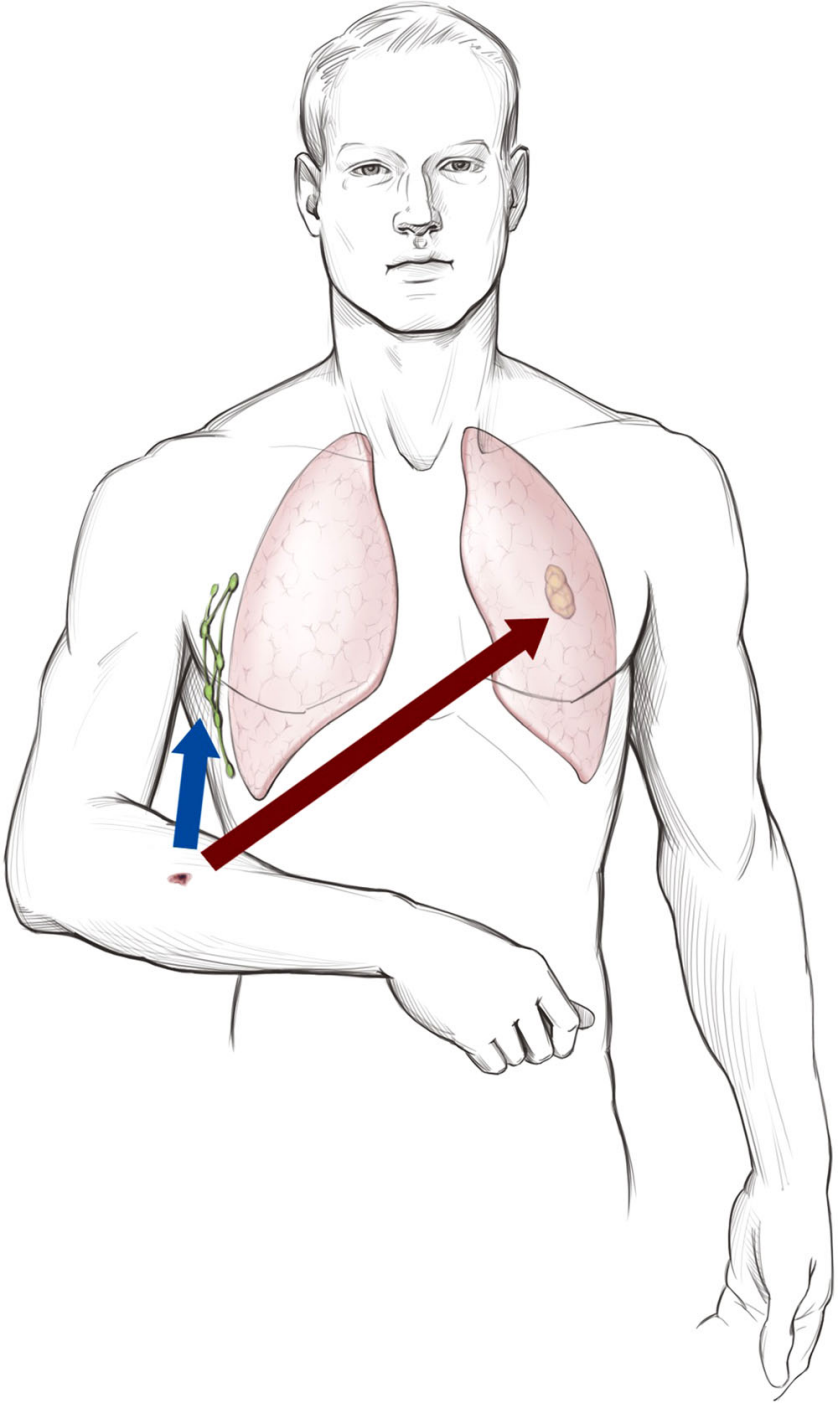
Potential outcomes in patients with melanoma undergoing sentinel lymph node biopsy, demonstrating hematogenous and lymphatic metastasis. Acknowledgement and credit to Brian C. Brockway, M.S., Medical Media, Atlanta VA Medical Center

Clinically, the tumor that we have the most experience with is cutaneous melanoma. Traditionally, sentinel lymph node biopsy has been offered to patients whose primary tumor is 1 mm or greater in thickness.^61^ Melanomas <1 mm in thickness are considered “thin melanomas” while melanomas thicker than 1 mm are considered “thick melanomas”. This is a distinction of clinical significance. The vast majority of melanomas <1 mm in diameter are cured by excision, and the rate of positive sentinel node metastasis in thinner melanomas is judged to be so low as not to risk the potential morbidity of lymph node biopsy.^62, 63^ However, it is a well-known phenomenon that some thin melanomas eventually present with distant metastasis. Today, we have no good biomarkers to determine which thin melanomas may undergo distant metastasis, although oncologists regard thin melanomas with mitoses and ulceration as potentially high risk for distant metastasis.^64^ Debate also exists on whether sentinel lymph node biopsy and exploration can be curative for a subset of patients. Sentinel lymph node biopsies are sometimes performed on patients with Spitz nevi, and lymph node spread is uncommon and removal of affected lymph nodes is curative.^45, 65^ Finally, it is universally acknowledged that lymph node positivity in cutaneous melanoma is an adverse prognostic factor.^56, 66^ How can all of these clinical observations be reconciled? Let us begin with the knowledge that the majority of melanomas arise as a result of driver mutations including Braf, Nras, and other less common mutations.^67–69^ Most melanomas under 1 mm thick contain cells capable of local invasion, but are not highly proliferative. Simple excision is curative in these cases. Thin melanomas that metastasize may contain small populations of metastasis-capable cells that have disseminated before the lesion is excised. In my own clinical experience, the presence of mitoses in thin melanomas increases the risk of distant metastasis. Thicker melanomas (>1 mm) have a high likelihood of containing populations of cells capable of both lymphatic metastasis and distant metastasis. A negative sentinel lymph node does not guarantee the freedom from distant metastasis, but positive sentinel lymph nodes indicate aggressive disease with both populations of lymphatic and distant metastasis.^62^ In such patients, lymphadenectomy might be useful for local control, but is not curative. There are thick melanomas that do not metastasize and there are melanomas that only metastasize to lymph nodes, but not distantly. Unfortunately, we do not have current biomarkers to assess this. However, ulceration of primary melanomas is associated with distant metastasis and tumor ulceration is often caused by high levels of reactive oxygen, increased secretion of matrix metalloproteinases, Akt, and angiopoietin-2.^70^ The potential outcomes in melanoma patients undergoing sentinel lymph node biopsy are depicted in Fig. 1.

## PGC1 alpha paradox—Why do tumor cells with respiratory function favor metastasis?

The Warburg effect, also named aerobic glycolysis, has long been known to be a feature of highly malignant cancer cells.^71, 72^ Advanced cancers inefficiently convert a single molecule of glucose into 2 molecules of ATP, unlike the 38 molecules of ATP that can be optimally derived from a molecule of glucose under full respiratory conditions. This is manifested by increased local concentrations of lactate, which may contribute to local tumor acidosis. We have hypothesized that the glycolytic cell gains a growth advantage in a hostile environment by activating NFκB.^40^ Indeed, most cells which exhibit aerobic glycolysis demonstrate NFκB activation, which prevents apoptosis in the hypoxic and acidotic tumor environment, and promotes resistance to chemotherapy and radiation, in part through upregulation of chemotherapy efflux pumps such as MDR. Cuezva et al.^72^ have shown that tumors of multiple organs with a high glycolytic index have a poor prognosis and have demonstrated that high-level expression of ATPase inhibitory factor 1 promotes glycolysis and tumorigenesis.

One would predict that, given inefficient ATP production from glucose, tumor cells would be starved. In fact, they are not. A marker of cellular starvation, AMPK activation, is not usually observed in tumor cells and, in fact, tumor cells are highly anabolic, as seen by activation of the target of rapamycin (mTORC1 and mTORC2) in a majority of malignant cells.^73, 74^ Tumor cells derive ATP and other raw materials from anabolism by increased glutamine uptake, beta oxidation of fatty acids, and macropinocytosis, which is commonly seen in ras-transformed cells and is inhibited by rac1 activation.^75, 76^ Thus, carcinogenesis is not an energy-deficient state, but a state of altered substrate use, which can be targeted. Indeed, an early chemotherapeutic modality is l-asparaginase, which targets the dependency of leukemia cells on the amino acid asparagine. More recently, Kim et al. have found that ceramide and analogs activate PP2A, which downregulates amino acids and lipid transporters, and thus can lead to a cancer cell-specific starvation.^77^ Of interest, reactive oxygen generated by NADPH oxidases can also activate PP2A.^78–80^

## Fission vs. fusion mitochondria

Mitochondrial function is governed at the level of transcription and protein composition. Normal respiratory mitochondria exist predominantly in an elongated morphology termed fusion mitochondria, while in highly malignant tumors, they exist in a fission morphology.^81, 82^ Mitochondria with fission may be less susceptible to mitochondrial apoptosis, mediated by VDAC-pore complexes,^83^ and may be a major reason for the Warburg phenomenon.^74^ Additional factors mediating mitochondrial metabolism are mitochondrial deacetylases, namely Sirt3, 4, and 5 and transcription of mitochondrial proteins mediated by the master transcriptional switch PGC1α, which forms a transcriptional complex with ERRα.^84^ Downstream targets of this complex include Nrf2, which transcriptionally activates a large family of enzymes involved in detoxifying cancer chemotherapeutics.^85, 86^ Mutations of Nrf2, which cause activation have been described in non-small-cell lung cancer, and are associated with mutant p53.^86, 87^ Of interest, Nrf2 activation promotes the rhoA/ROCK signaling pathway in breast cancer cells. This is what would be expected in mutant p53 cancers with low levels of reactive oxygen species.^88^

Recently, elevated PGC1α has been found to be a negative prognostic factor in cutaneous melanoma and other tumors.^49, 89^ Patients with high levels of PGC1α were observed to have shortened survival, presumably due to metastasis. Similar findings have been seen in lapatinib resistance in breast cancer due to increased expression of ERRα. Conversely, low levels of PGC1α are seen in vertical growth melanoma.^90^ How can we reconcile these seemingly contradictory findings? On the one hand, there is increased death due to high expression of PGC1α in melanoma; on the other hand, PGC1α expression is decreased in vertical growth melanoma. This phenomenon is likely not limited to melanoma. In order to better understand this phenomenon, I communicated with Dr. Pere Puigserver, who reports that, in a comparison between advanced melanomas, high levels of PGC1α are a poor prognostic factor. However, in early melanoma, PGC1α is highly expressed, leading to a model of plasticity in which early melanoma cells (at least the subset that highly expresses PGC1α) need to downregulate PGC1α to migrate and disseminate. In fact, PGC1α correlates with radial, but not vertical, growth in melanomas (Pere Puigserver, personal communications 2016). This would imply that there might be a population of cells with impaired mitochondrial function that migrate away from a central tumor to distant sites and, once reaching a favorable niche, re-expresses PGC1α and forms a metastasis.

**Fig. 2:**
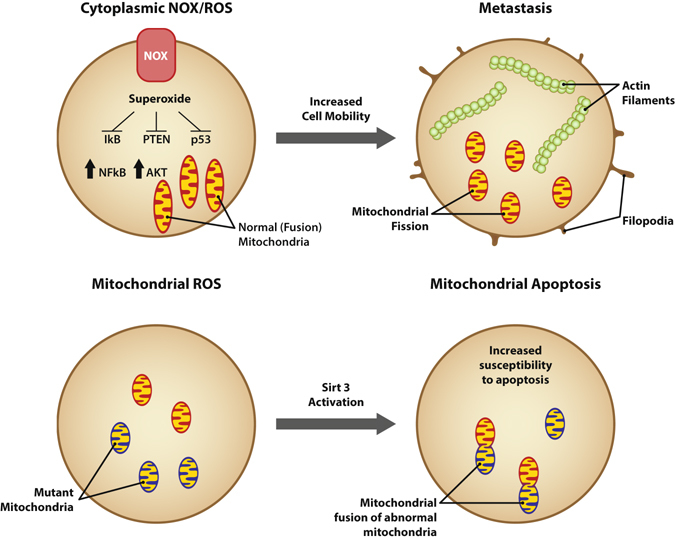
Signaling in primary tumor and metastasis based upon mitochondrial bioenergetic profile. Large primary tumors express high levels of PCG1α in a component of their cells. Respiratory cells have both motile and non-motile components. Non-motile components have increased susceptibility to chemotherapy and radiation and represent, in part, the initial population that responds to chemotherapy and radiation. After maximal response, a glycolytic slow growing population is left behind, as well as motile respiratory cells that give rise to metastasis. These metastatic cells may maintain respiration through elevated levels of ClpXP. Once metastatic cells reach a niche, they can reform a mixed population of respiratory and glycolytic cells. Acknowledgement and credit to Brian C. Brockway, M.S., Medical Media, Atlanta VA Medical Center

Thus, it is likely that primary tumors contain mixed populations of low and high PGC1α-expressing cells. Tumors with elevated PGC1α might be more susceptible to chemotherapy and radiation, accounting for the initial decrease in tumor size. Residual cells left behind after chemotherapy might be a mixed population of low PGC1α-expressing cells, some of which are motile and give rise to metastasis, and some of which are stationary, which then re-express PGC1α and regrow, causing recurrence (Fig. 2). Of interest, the Mitochondrial Unfoldase–Peptidase Complex ClpXP recently discovered to be a survivin binding partner, is required for both tumor cell respiration and migration. Thus, it is a possibility that the metastasizing tumor cell has a low PGC1α expression, which might be compensated by elevated levels of ClpXP that would allow respiration in metastasizing tumor cells.^91^ Intriguingly, a small molecule Sirt3 activator, honokiol,^92^ has several similar activities to knockdown of ClpXP, including decreased metastatic ability, induction of AMP kinase, decreased survivin, induction of succinate dehydrogenase, and decrease in PGC1α expression.^73, 93–95^ It is thus likely that tumor cell respiration requires ClpXP. High-level expression of ClpXP may be one of the factors that allows tumor cells to undergo respiration, but is not necessarily required for normal cell respiration.

**Fig. 3:**
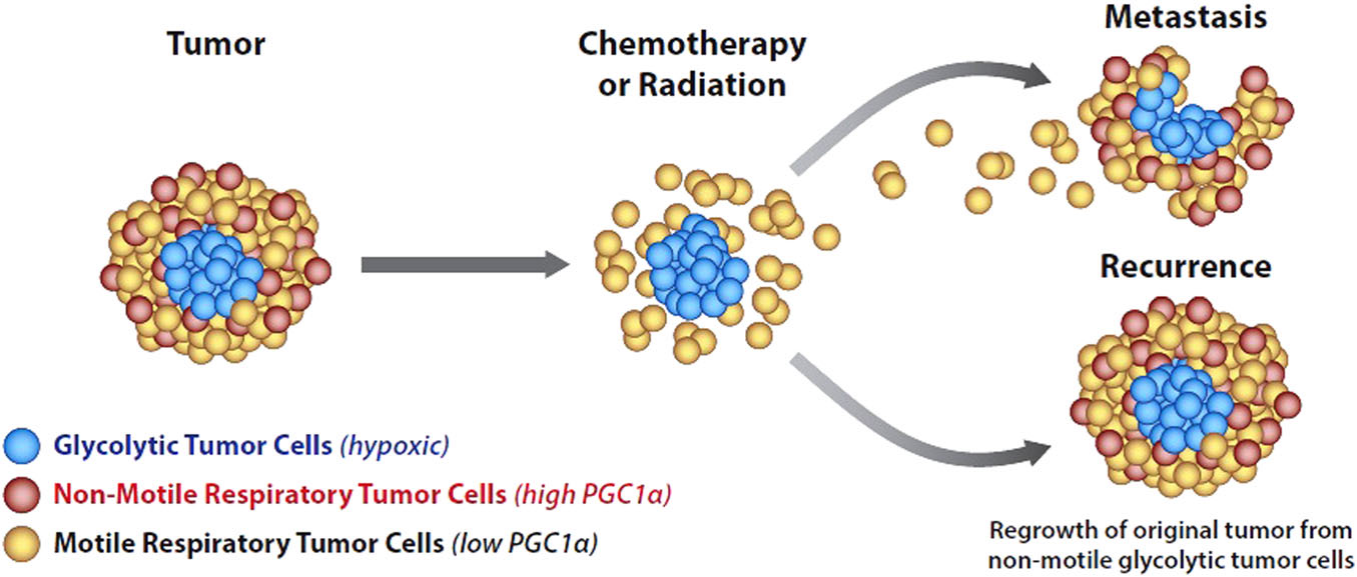
The source and location of reactive oxygen plays a dualistic role in tumor cells. NADPH oxidases are tethered to the cellular membrane in part by Akt. These complexes generate superoxide that oxidizes redox-sensitive sulfhydryl groups in the cytoplasm. This leads to inactivation of cellular phosphatases, activation of cellular kinases, and polymerization of F-actin, allowing motility. In contrast, mitochondrial-derived reactive oxygen may be a highly pro-apoptotic mechanism, leading to VDAC channel formation, loss of mitochondrial potential and, thus, loss of ATP. Fusion mitochondria may be highly susceptible to these events, while fission mitochondria may be less susceptible to these events, and fission may serve as a defense mechanism against mitochondrial reactive oxygen. Acknowledgement and credit to Brian C. Brockway, M.S., Medical Media, Atlanta VA Medical Center

Location, location, location! The most important factor in real estate likely plays a similarly important role in tumor cell function. Cytoplasmic superoxide generation mediated by NADPH oxidase complexes usually exerts pro-tumorigenic effects by oxidizing cytoplasmic phosphatases such as IκB, PTEN, and PP2a and, thus, activating downstream kinases. On the other hand, mitochondrial reactive oxygen often mediates mitochondrial apoptosis and death of tumor cells. It remains to be determined whether fission mitochondria in tumor cells provide a defense against mitochondrial apoptosis (Fig. 3).

## Conclusion

Representing duality in cancer is an oversimplification, yet it is a useful model for the development of novel treatments for cancer. Many solid tumors have a tremendous mutational burden, and it is impractical at the current time to sequence every patients tumor and target all the mutations that exist in a given patients tumor. For those studying cancer, it is useful to recognize that given driver mutations function differently in tumors with mutant p53 and loss of p16ink4a. It is also important to recognize that even in a single tumor with a given p53 vs. p16 status, there is plasticity in mitochondrial function and cellular signaling. This is often clinically manifested in metastatic disease. For example, an aggressive primary melanoma or breast cancer can give rise to both lymphatic disease and distant disease. This means that the primary tumor has cells that use rhoA/ROCK signaling and undergo ameboid metastasis to lymphatics, as well as cells that use reactive oxygen/Akt signaling and undergo mesenchymal hematogenous metastasis to distant sites. The plasticity also means that treatment of a tumor may synchronize a plastic tumor to a particular pole of signaling. For instance, reactive oxygen-causing therapies, such as radiation therapy and certain cytotoxic chemotherapies, may select for existing populations of tumor cells that are highly radiation resistant because they use superoxide to activate NFκB and Akt. Synchronization may also lead to more effective sequential therapies to eliminate tumors when they have been polarized to a given signaling pathway. Mitochondrial polarization between glycolysis (fission) and respiration (fusion) also plays a role in response to therapy. Much evidence now suggests that primary solid tumors contain glycolytic (Warburg) cells that are resistant to hypoxia, chemotherapy, and radiation, and are slow growing, admixed with more responsive and more rapidly growing respiratory cells. Similarly, respiratory cells can be divided into non-motile tumor cells that contribute to tumor mass and motile tumor cells, which metastasize. Respiratory tumor cells carry unique vulnerabilities that can distinguish them from normal respiratory cells, such as defective mitochondria and decreased NFκB that could be targeted by therapies that include Sirt3 activation, which kills tumor cells with defective mitochondria by promotion of mitochondrial fusion and increased reactive oxygen generation, while, at the same time, decreasing NFκB activation. A more widespread understanding of these facts by both researchers and clinicians will lead to novel treatments with enhanced efficacy.

## References

[1] Pardoll DM (2012). The blockade of immune checkpoints in cancer immunotherapy. Nat. Rev. Cancer.

[2] Kalthoff H (1993). p53 and K-RAS alterations in pancreatic epithelial cell lesions. Oncogene.

[3] Berrozpe G, Schaeffer J, Peinado MA, Real FX, Perucho M (1994). Comparative analysis of mutations in the p53 and K-ras genes in pancreatic cancer. Int. J. Cancer..

[4] Klangby U (1998). p16/INK4a and p15/INK4b gene methylation and absence of p16/INK4a mRNA and protein expression in Burkitt’s lymphoma. Blood..

[5] Arbiser JL (2004). Involvement of p53 and p16 tumor suppressor genes in recessive dystrophic epidermolysis bullosa-associated squamous cell carcinoma. J. Invest. Dermatol..

[6] Moskaluk CA, Hruban RH, Kern SE (1997). p16 and K-ras gene mutations in the intraductal precursors of human pancreatic adenocarcinoma. Cancer Res..

[7] LaMontagne KR (2000). Inhibition of MAP kinase kinase causes morphological reversion and dissociation between soft agar growth and in vivo tumorigenesis in angiosarcoma cells. Am. J. Pathol..

[8] Govindarajan B (2002). Reactive oxygen-induced carcinogenesis causes hypermethylation of p16(Ink4a) and activation of MAP kinase. Mol. Med..

[9] Cerimele F (2005). Reactive oxygen signaling and MAPK activation distinguish Epstein-Barr Virus (EBV)-positive versus EBV-negative Burkitt’s lymphoma. Proc. Natl Acad. Sci. USA..

[10] Kamb A (1994). Analysis of the p16 gene (*CDKN2*) as a candidate for the chromosome 9p melanoma susceptibility locus. Nat. Genet..

[11] Church SL (1993). Increased manganese superoxide dismutase expression suppresses the malignant phenotype of human melanoma cells. Proc. Natl Acad. Sci. USA..

[12] Fried L, Arbiser JL (2008). The reactive oxygen-driven tumor: relevance to melanoma. Pigment Cell Melanoma Res..

[13] Proweller A (2006). Impaired notch signaling promotes *de novo* squamous cell carcinoma formation. Cancer Res..

[14] Oberholzer PA (2012). RAS mutations are associated with the development of cutaneous squamous cell tumors in patients treated with RAF inhibitors. J. Clin. Oncol..

[15] Martincorena I (2015). Tumor evolution. High burden and pervasive positive selection of somatic mutations in normal human skin. Science.

[16] Pierceall WE, Mukhopadhyay T, Goldberg LH, Ananthaswamy HN (1991). Mutations in the p53 tumor suppressor gene in human cutaneous squamous cell carcinomas. Mol. Carcinog..

[17] Mulligan LM, Matlashewski GJ, Scrable HJ, Cavenee WK (1990). Mechanisms of p53 loss in human sarcomas. Proc. Natl Acad. Sci. USA..

[18] Arbiser JL (1997). Oncogenic H-ras stimulates tumor angiogenesis by two distinct pathways. Proc. Natl Acad. Sci. USA..

[19] Quelle DE (1995). Cloning and characterization of murine p16INK4a and p15INK4b genes. Oncogene.

[20] Kim HS (2010). SIRT3 is a mitochondria-localized tumor suppressor required for maintenance of mitochondrial integrity and metabolism during stress. Cancer Cell.

[21] Lebok P (2016). p16 overexpression and 9p21 deletion are linked to unfavorable tumor phenotype in breast cancer. Oncotarget.

[22] Moriyama T (2007). Hypermethylation of p14 (ARF) may be predictive of colitic cancer in patients with ulcerative colitis. Dis. Colon Rectum.

[23] Jiao L (2007). K-ras mutation and p16 and preproenkephalin promoter hypermethylation in plasma DNA of pancreatic cancer patients: in relation to cigarette smoking. Pancreas..

[24] Gutierrez MI (2004). CpG island methylation in Schistosoma- and non-Schistosoma-associated bladder cancer. Mod. Pathol..

[25] Shah SP (2012). The clonal and mutational evolution spectrum of primary triple-negative breast cancers. Nature..

[26] Curtis C (2012). The genomic and transcriptomic architecture of 2,000 breast tumours reveals novel subgroups. Nature..

[27] Jimenez RE (1999). Sequential accumulation of K-ras mutations and p53 overexpression in the progression of pancreatic mucinous cystic neoplasms to malignancy. Ann. Surg..

[28] Kang KA, Zhang R, Kim GY, Bae SC, Hyun JW (2012). Epigenetic changes induced by oxidative stress in colorectal cancer cells: methylation of tumor suppressor RUNX3. Tumour Biol..

[29] Mishra MV (2008). DNMT1 as a molecular target in a multimodality-resistant phenotype in tumor cells. Mol. Cancer Res..

[30] Arbiser JL (2006). Presence of p16 hypermethylation and Epstein-Barr virus infection in transplant-associated hematolymphoid neoplasm of the skin. J. Am. Acad. Dermatol..

[31] Bhandarkar SS, MacKelfresh J, Fried L, Arbiser JL (2008). Targeted therapy of oral hairy leukoplakia with gentian violet. J. Am. Acad. Dermatol..

[32] Perry BN (2006). Pharmacologic blockade of angiopoietin-2 is efficacious against model hemangiomas in mice. J. Invest. Dermatol..

[33] Jonason AS (1996). Frequent clones of p53-mutated keratinocytes in normal human skin. Proc. Natl Acad. Sci. USA.

[34] Bonner MY, Arbiser JL (2012). Targeting NADPH oxidases for the treatment of cancer and inflammation. Cell. Mol. Life Sci..

[35] Wong L, Zhou J, Anderson D, Kratzke RA (2002). Inactivation of p16INK4a expression in malignant mesothelioma by methylation. Lung Cancer.

[36] Pollock PM (2003). High frequency of BRAF mutations in nevi. Nat. Genet..

[37] Cohen C (2002). Mitogen-actived protein kinase activation is an early event in melanoma progression. Clin. Cancer Res..

[38] Govindarajan B (2003). Malignant transformation of melanocytes to melanoma by constitutive activation of mitogen-activated protein kinase kinase (MAPKK) signaling. J. Biol. Chem..

[39] Boivin B, Zhang S, Arbiser JL, Zhang ZY, Tonks NK (2008). A modified cysteinyl-labeling assay reveals reversible oxidation of protein tyrosine phosphatases in angiomyolipoma cells. Proc. Natl Acad. Sci. USA..

[40] Govindarajan B (2007). Overexpression of Akt converts radial growth melanoma to vertical growth melanoma. J. Clin. Invest..

[41] Arbiser JL, Bips M, Seidler A, Bonner MY, Kovach C (2012). Combination therapy of imiquimod and gentian violet for cutaneous melanoma metastases. J. Am. Acad. Dermatol..

[42] Pietrobono S (2016). Down-regulation of SOX2 underlies the inhibitory effects of the triphenylmethane gentian violet on melanoma cell self-renewal and survival. J. Invest. Dermatol..

[43] Nonaka D (2009). Differential expression of SOX2 and SOX17 in testicular germ cell tumors. Am. J. Clin. Pathol..

[44] Li AS (2008). Hypermethylation of *SOX2* gene in hydatidiform mole and choriocarcinoma. Reprod. Sci..

[45] Barnhill RL, Flotte TJ, Fleischli M, Perez-Atayde A (1995). Cutaneous melanoma and atypical Spitz tumors in childhood. Cancer.

[46] Lallas A (2014). Atypical Spitz tumours and sentinel lymph node biopsy: a systematic review. Lancet Oncol..

[47] Herbst A, Haynes HA, Nghiem P (2002). The standard of care for Merkel cell carcinoma should include adjuvant radiation and lymph node surgery. J. Am. Acad. Dermatol..

[48] Nghiem P (2015). Merkel cell carcinoma: intersection of immune dysfunction, infection, and malignant progression. J. Investig. Dermatol. Symp. Proc..

[49] LeBleu VS (2014). PGC-1alpha mediates mitochondrial biogenesis and oxidative phosphorylation in cancer cells to promote metastasis. Nat. Cell. Biol..

[50] Parri M, Chiarugi P (2010). Rac and Rho GTPases in cancer cell motility control. Cell Commun. Signal..

[51] Parri M, Taddei ML, Bianchini F, Calorini L, Chiarugi P (2009). EphA2 reexpression prompts invasion of melanoma cells shifting from mesenchymal to amoeboid-like motility style. Cancer Res..

[52] Sanz-Moreno V (2008). Rac activation and inactivation control plasticity of tumor cell movement. Cell.

[53] Hirono I (1958). Ameboid motility of the ascites hepatoma cells and its significance for their invasiveness and metastatic spread. Cancer Res..

[54] Amin AR (2015). Evasion of anti-growth signaling: a key step in tumorigenesis and potential target for treatment and prophylaxis by natural compounds. Semin. Cancer Biol..

[55] Ahmed M (2016). An osteopontin/CD44 axis in RhoGDI2-mediated metastasis suppression. Cancer Cell.

[56] Balch CM (2014). Age as a predictor of sentinel node metastasis among patients with localized melanoma: an inverse correlation of melanoma mortality and incidence of sentinel node metastasis among young and old patients. Ann. Surg. Oncol..

[57] Gallagher SJ (2013). Beta-catenin inhibits melanocyte migration but induces melanoma metastasis. Oncogene..

[58] Crespo P, Calvo F, Sanz-Moreno V (2011). Ras and Rho GTPases on the move: the RasGRF connection. Bioarchitecture.

[59] Balch CM (2010). Multivariate analysis of prognostic factors among 2,313 patients with stage III melanoma: comparison of nodal micrometastases versus macrometastases. J. Clin. Oncol..

[60] Zhuge Y, Xu J (2001). Rac1 mediates type I collagen-dependent MMP-2 activation: role in cell invasion across collagen barrier. J. Biol. Chem..

[61] O’Reilly FM (2001). Microphthalmia transcription factor immunohistochemistry: a useful diagnostic marker in the diagnosis and detection of cutaneous melanoma, sentinel lymph node metastases, and extracutaneous melanocytic neoplasms. J. Am. Acad. Dermatol..

[62] Han D (2012). Sentinel node biopsy is indicated for thin melanomas>/=0.76 mm. Ann. Surg. Oncol..

[63] Morton DL (2006). Sentinel-node biopsy or nodal observation in melanoma. N. Engl. J. Med..

[64] Soong SJ (2010). Predicting survival outcome of localized melanoma: an electronic prediction tool based on the AJCC melanoma database. Ann. Surg. Oncol..

[65] Arbiser JL (1996). Angiogenesis and the skin: a primer. J. Am. Acad. Dermatol..

[66] Balch CM (2013). Age as a prognostic factor in patients with localized melanoma and regional metastases. Ann. Surg. Oncol..

[67] Krauthammer M (2015). Exome sequencing identifies recurrent mutations in NF1 and RASopathy genes in sun-exposed melanomas. Nat. Genet..

[68] Krauthammer M (2012). Exome sequencing identifies recurrent somatic RAC1 mutations in melanoma. Nat. Genet..

[69] Tsao H, Zhang X, Fowlkes K, Haluska FG (2000). Relative reciprocity of NRAS and PTEN/MMAC1 alterations in cutaneous melanoma cell lines. Cancer Res..

[70] Niessner H (2013). Targeting hyperactivation of the AKT survival pathway to overcome therapy resistance of melanoma brain metastases. Cancer Med..

[71] Warburg O (1956). On respiratory impairment in cancer cells. Science.

[72] Cuezva JM (2002). The bioenergetic signature of cancer: a marker of tumor progression. Cancer Res..

[73] Nagalingam A, Arbiser JL, Bonner MY, Saxena NK, Sharma D (2012). Honokiol activates AMP-activated protein kinase in breast cancer cells via an LKB1-dependent pathway and inhibits breast carcinogenesis. Breast Cancer Res..

[74] Vyas S, Zaganjor E, Haigis MC (2016). Mitochondria and Cancer. Cell.

[75] Commisso C (2013). Macropinocytosis of protein is an amino acid supply route in Ras-transformed cells. Nature..

[76] Fujii M, Kawai K, Egami Y, Araki N (2013). Dissecting the roles of Rac1 activation and deactivation in macropinocytosis using microscopic photo-manipulation. Sci. Rep..

[77] Kim SM (2016). Targeting cancer metabolism by simultaneously disrupting parallel nutrient access pathways. J. Clin. Invest..

[78] Sanchez-Sanchez B (2014). NADPH oxidases as therapeutic targets in chronic myelogenous leukemia. Clin. Cancer Res..

[79] Laidlaw KM (2016). Cooperation of imipramine blue and tyrosine kinase blockade demonstrates activity against chronic myeloid leukemia. Oncotarget.

[80] Nieborowska-Skorska M, Flis S, Skorski T (2014). AKT-induced reactive oxygen species generate imatinib-resistant clones emerging from chronic myeloid leukemia progenitor cells. Leukemia.

[81] Rehman J (2012). Inhibition of mitochondrial fission prevents cell cycle progression in lung cancer. FASEB J..

[82] Serasinghe MN (2015). Mitochondrial division is requisite to RAS-induced transformation and targeted by oncogenic MAPK pathway inhibitors. Mol. Cell.

[83] Zaid H, Abu-Hamad S, Israelson A, Nathan I, Shoshan-Barmatz V (2005). The voltage-dependent anion channel-1 modulates apoptotic cell death. Cell Death Differ..

[84] Arany Z (2008). HIF-independent regulation of VEGF and angiogenesis by the transcriptional coactivator PGC-1alpha. Nature.

[85] Jiang T (2010). High levels of Nrf2 determine chemoresistance in type II endometrial cancer. Cancer Res..

[86] Jeong, Y. et al. Role of KEAP1/NRF2 and TP53 mutations in lung squamous cell carcinoma development and radiation resistance. *Cancer Discov*. doi:10.1158/2159-8290.CD-16-0127 (2016). 10.1158/2159-8290.CD-16-0127 PMC5222718 27663899

[87] Tung MC (2015). Mutant p53 confers chemoresistance in non-small cell lung cancer by upregulating Nrf2. Oncotarget.

[88] Zhang, C. et al. NRF2 promotes breast cancer cell proliferation and metastasis by increasing RhoA/ROCK pathway signal transduction. *Oncotarget* doi:10.18632/oncotarget.12435 (2016). 10.18632/oncotarget.12435 PMC5342001 27713154

[89] Vazquez F (2013). PGC1alpha expression defines a subset of human melanoma tumors with increased mitochondrial capacity and resistance to oxidative stress. Cancer Cell.

[90] Luo C (2016). A PGC1alpha-mediated transcriptional axis suppresses melanoma metastasis. Nature.

[91] Seo JH (2016). The mitochondrial unfoldase-peptidase complex ClpXP controls bioenergetics stress and metastasis. PLoS Biol..

[92] Pillai VB (2015). Honokiol blocks and reverses cardiac hypertrophy in mice by activating mitochondrial Sirt3. Nat. Commun..

[93] Steinmann P (2012). Antimetastatic activity of honokiol in osteosarcoma. Cancer.

[94] Medra, A. et al. Pro-apoptotic activity of new honokiol/triphenylmethane analogues in B-cell lymphoid malignancies. *Molecules*. **21**, doi:10.3390/molecules21080995 (2016). 10.3390/molecules21080995 PMC6274336 27483232

[95] Ahn KS (2006). Honokiol potentiates apoptosis, suppresses osteoclastogenesis, and inhibits invasion through modulation of nuclear factor-kappaB activation pathway. Mol. Cancer Res..

